# Homocysteine and cognitive impairment; a case series in a General Practice setting

**DOI:** 10.1186/1475-2891-5-6

**Published:** 2006-02-15

**Authors:** Andrew McCaddon

**Affiliations:** 1 Honorary Research Fellow, Wales College of Medicine, Wrexham, Uk

## Abstract

**Background:**

An elevated blood level of homocysteine is a risk factor for cognitive impairment and dementia. Homocysteine can be lowered by folate and/or vitamin B_12 _supplementation; antioxidants might also be required for optimal reduction in neurovascular tissue. This report presents clinical and radiological findings from administering the antioxidant N-acetylcysteine together with B vitamins to cognitively impaired patients with hyperhomocysteinaemia.

**Methods:**

A case series (n = 7) performed in a semi-rural General Practice setting. Formal cognitive assessments were performed in five patients, and radiological assessments in one patient, before and after supplementation.

**Results and discussion:**

The addition of N-acetylcysteine resulted in subjective clinical improvement in all patients, and an objective improvement in cognitive scores in five patients. One patient had radiological evidence of halted disease progression over a twelve month period.

**Conclusion:**

N-acetylcysteine, together with B vitamin supplements, improves cognitive status in hyperhomocysteinaemic patients. Randomized controlled clinical trials are required to formally evaluate this treatment approach.

## Background

Vitamin B_12 _is essential for two mammalian metabolic reactions – the conversion of methylmalonyl-CoA to succinyl-CoA, and of homocysteine to methionine; the latter reaction is also folate dependent [1]. Serum levels of methylmalonic acid rise in B_12 _deficiency, whereas homocysteine levels rise in both folate *and *B_12 _deficiency. The advent of assays for these metabolites has facilitated the detection of early and 'subtle' deficiencies of these vitamins [2].

Of these two metabolites, homocysteine has recently attracted interest with regard to cognitive function and aging. Elevated plasma homocysteine is associated with an increased risk of vascular disease and vascular dementia [3,4]. There are also widely confirmed reports of elevated blood levels in patients with Alzheimer's disease (AD) and mild cognitive impairment (MCI) [5-8]. Homocysteine levels predict cognitive decline in healthy elderly [9-11], and hyperhomocysteinaemia is an independent risk factor for the development of dementia, including AD [12,13]. However, not all studies confirm a relationship between homocysteine and cognition [14-17].

Potential mechanisms by which homocysteine might influence cognition include a direct toxicity on glutamate neurotransmission and cerebrovascular endothelium, an indirect inhibition of transmethylation reactions in brain, potentiation of amyloid neurotoxicity and promotion of tau phosphorylation [18-20].

Such mechanisms suggest causality, but confirmation requires evidence from randomized controlled intervention trials. Three such trials are now underway. The VITAL trial (VITamins to slow ALzheimer's disease) is a large (n = 400) double-blind placebo-controlled trial to determine whether high-dose B vitamin supplements slow cognitive decline in subjects with established AD [21]. The VITATOPS (VITAmins TO Prevent Stroke) study is a large (n = 8,000) multi-center, randomized, double blind, placebo-controlled secondary stroke prevention trial to determine whether the addition of B-supplements to current best management reduces the incidence of recurrent vascular events in patients with stroke or transient ischaemic attacks; dementia is a secondary outcome measure [22]. VITACOG is a UK-based randomized placebo-controlled trial of B-supplements in 300 elderly participants with MCI to determine effects on brain atrophy and cognitive function (AD Smith – personal communication).

Although plasma levels of homocysteine are largely determined by vitamin B_12 _and folate status, antioxidant therapy might also be required for optimal reduction in neurovascular tissue [23]. This report presents the effects of administering the antioxidant N-acetylcysteine (NAC) together with B vitamins in seven cognitively impaired patients presenting to their General Practitioner (AMc) with hyperhomocysteinaemia and/or B vitamin deficiency. Patients and carers were informed of the rationale for prescribing NAC, and of its use outside the scope of its current product licence, in accordance with United Kingdom General Medical Council Guidelines [24].

## Case presentations

### Case 1

A 78 year old lady presented with fatigue, anxiety and depression. She had angular cheilosis but was not anaemic (Hb 11.9, MCV 90), despite a profound vitamin B_12 _deficiency (11 ng/l) with normal serum and red cell folate (12 μg/l and 299 μg/L). She had parietal cell antibodies but a normal Schilling test. She '...felt better' after monthly injections of hydroxo-B_12 _(1,000 μg) were commenced, but nevertheless remained anxious.

A year later she developed memory impairment, with difficulty remembering names. She scored 21/30 on Mini-mental state examination (MMSE) [25]. It was felt that she had a dementia, perhaps with associated depression, and she was commenced on an antidepressant (a selective serotonin reuptake inhibitor). Her depression slowly resolved but her cognitive decline continued.

A year later she scored 18/30 on MMSE and 28/70 on the Alzheimer's disease assessment scale (ADAS-Cog) [26]. In view of her persistent dementia despite regular hydroxo-B_12 _injections, she was commenced on oral NAC (600 mg) daily *(Zambon Italia – Vicenza)*. Two weeks later her husband reported a noticeable improvement in her memory. She now remembered names and faces she previously would have struggled to recall. Her MMSE improved to 21/30 and ADAS-Cog to 20/70. The areas of improvement were in scores of orientation, copying skills, word-recall, naming and commands.

### Case 2

An 84 year old lady presented with a three year history of short-term memory impairment and early Parkinsonism for which she took L-dopa. She was otherwise well. She scored 12/28 on the 6 Item Cognitive Impairment Test (6CIT) [27]. Investigations revealed a total serum homocysteine (tHcy) of 20.1 μmol/L (normal range <13μmol/L), but normal serum vitamin B_12 _(309 ng/L), folate (9.2 μg/L) and red cell folate (464 μg/L). She was not anaemic, but had borderline hypothyroidism (TSH 9.1 μmol/L).

In view of her elevated tHcy, and despite 'normal' serum B vitamin levels, she was commenced on daily oral cyano-B_12 _(1000 μcg), folic acid (5 mg) and NAC (600 mg). Within one month her tHcy fell to 7.5 μmol/L. She was assessed by a Psychogeriatrician three months after her initial presentation. She now showed no significant cognitive deficits; she scored 28/30 on MMSE.

The psychogeriatrician was '...particularly impressed that she could provide the Christian names of all her five children and twelve grandchildren without any problems' and it was felt that she now had no obvious diagnosis of a dementing illness.

### Case 3

A 77 year old lady presented with a six month history of confusion and memory loss. An aunt had early-onset AD. On examination she was disorientated to time and had demonstrable memory impairment. She was vitamin B_12 _deficient (172 ng/L) but with normal serum and red cell folate (10.1 μg/L and 321 μg/l, respectively).

She commenced monthly intramuscular hydroxo-B_12 _injections (1,000 μg) but continued to deteriorate. She became fatigued and developed visual hallucinations and persecutory ideas. She had naming difficulties, her repetition was poor, and she had constructional dyspraxia. It was felt she had probable AD. She was admitted for care, scoring only 13/30 on MMSE. She was commenced on an acetylcholinesterase inhibitor with slight initial improvement (15/30). Nevertheless her condition continued to deteriorate. She developed dysphagia and weight loss due to a grade III oesophagitis.

Oral NAC (600 mg daily) was added to her treatment and her family and carers noticed a significant improvement. She became more alert and recognised her close family; a formal cognitive assessment was not performed in view of the severity of her dementia and associated physical condition. Sadly she died from a bronchopneumonia several weeks later.

### Case 4

An 87 year old retired school headmistress presented with a three year history of gradually deteriorating short-term memory and general 'confusion'; she frequently mislaid things and often wandered, forgetting her way home. She had a past medical history of diverticular disease and an osteoarthritic hip, but was otherwise well.

On examination it was felt she was suffering from a senile dementia of moderate severity although no formal cognitive scores were recorded. Routine investigations were normal other than a highly elevated tHcy of 27.5 μmol/L.

She was commenced on folic acid 5 mg daily and oral hydroxo-B_12 _1,000 mcg daily, together with NAC 600 mg daily. Her tHcy fell to 6.6 μmol/L six-months later, at which time she felt generally well although she remained mildly confused. Her daughter commented on a marked improvement in her general behaviour, although she remained forgetful at times. Three years later she remains very well and continues to be cared for at home with no major difficulties.

### Case 5

An 84 year old lady presented with a two year history of increasing forgetfulness and confusion and no significant previous illnesses. She was cared for at home by her daughter. On formal examination she was disorientated in time and place with demonstrable short-term memory impairment.

It was felt she had a moderate dementia; she scored 55 out of 70 on an ADAS-Cog assessment. Blood investigations were normal other than an elevated tHcy of 16.4 μmol/L and low red cell folate (157μg/l) (serum vitamin B_12 _and folate were 281 ng/L and 4.4 μg/l respectively.) She was not anaemic (Hb 12.7).

She was commenced on daily oral cyano-B_12 _(150 μg daily) folic acid (5 mg) and NAC (600 mg) daily. One month later she had gained ten points on ADAS-Cog score. Her daughter also reported that she was '...generally more settled and content, and less likely to wander.'

### Case 6

A 71 year old retired engineer presented with a ten year history of gradually progressive short-term memory impairment. His wife had become concerned because he had recently lost his way driving to a regular address. He complained of losing objects and forgetting people's names.

His father had died of senile dementia aged 74, with symptoms developing at aged 60 years. The patient himself had a past medical history of hypertension controlled by beta-blocker. On cognitive examination he scored poorly on 6CIT (12/28). Routine blood investigations revealed elevated tHcy (15.6 μmol/L) with normal serum vitamin B_12 _(368 ng/L), folate (9.3μg/L) and red cell folate (492 μg/L).

He was commenced on daily oral cyano-B_12 _(1,000 μg) folate (5 mg) and NAC (600 mg). His wife reported a "...definite and immediate improvement" within two weeks of commencing treatment. His tHcy fell to 9.6 μmol/L. He was seen by a Psychogeriatrician one month later. He now scored 28/30 on MMSE with a CAMCOG of 115/125, indicating only a mild cognitive deficit [28]. A diagnosis of "age-related cognitive impairment" was made. He has continued on this treatment for the last two years and continues to score well on cognitive assessments. He scored 27/30 on his most recent assessment and has now commenced an acetylcholinesterase inhibitor in addition to B vitamin and NAC supplementation.

### Case 7

A 75 year old retired College Lecturer presented with a four year history of increasing forgetfulness. In particular he frequently mislaid things, and had occasional difficulty remembering people's names. His wife had become concerned about his ability to drive. He had a past medical history of prostatic carcinoma treated with radiotherapy, anxiety/depression, and gastro-oesophageal reflux, but was generally well in himself.

**Figure 1:**
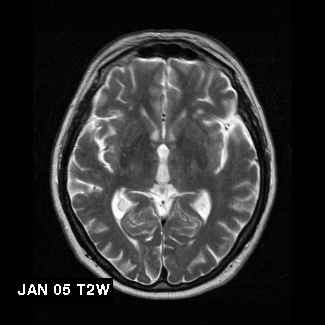
Initial radiological findings in Case 7: T2 weighted MRI scans of Case 7 showing extent of white matter disease in January 2005

On cognitive examination he was disorientated in time and had demonstrable short term memory impairment. He scored 8/28 on 6CIT and 16/39 on TICS-m [29]. An MRI scan showed several foci consistent with small vessel disease (Figure 1). Blood investigations revealed raised tHcy of 14.6 μmol/L, borderline low serum B_12 _(191 ng/L), but normal folate (11.7 μg/L) and red cell folate (692 μg/L).

He was commenced on daily oral cyano-B_12 _(1000 μg), folic acid (5 mg) and NAC (600 mg). After one month he had gained five points on TICS-m (21/39). His tHcy fell to 8.3 μmol/L. He was personally delighted with the treatment. His wife commented that "...he was becoming very forgetful, quite retiring, quiet, sleepy all the time, not interested in his food or in life. Now, there's a marked improvement. We can discuss the news without any problem. He's reading again. He's interested in life once more."

**Figure 2:**
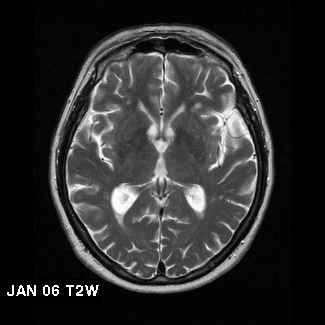
Follow-up radiological findings in Case 7: T2 weighted MRI scans of Case 7 showing extent of white matter disease in January 2006

One year later, he remains well and has successfully regained his driving license following a formal assessment. A repeat MRI scan showed no significant progression in the extent or size of the focal areas of abnormality in the deep white matter, and no change in ventricular configuration (Figure 2).

## Conclusion

These reports demonstrate the apparent clinical efficacy of the addition of NAC to B vitamin regimes in hyperhomocysteinaemic patients with cognitive impairment. NAC was well-tolerated in all patients; there were no reported side-effects. Three similar case reports were described earlier [30]. There is one previous study of NAC treatment alone in AD patients [31]. This found a favourable effect of NAC on nearly every outcome measure, although significant differences were obtained only for a subset of cognitive tasks.

There is now strong evidence that elevated blood levels of homocysteine are associated with dementia in general, including both vascular dementia and AD [32]. However, the mechanism underlying this association remains unclear. It may be due to a combination of adverse affects of homocysteine on neurovascular tissue, in addition to impairment of neurotransmitter synthesis due to defects in methyl group metabolism [19,20]. The clinical responses to NAC in these cases suggest that homocysteine might also be a surrogate marker for the effects of oxidative stress in these tissues [23].

**Figure 3:**
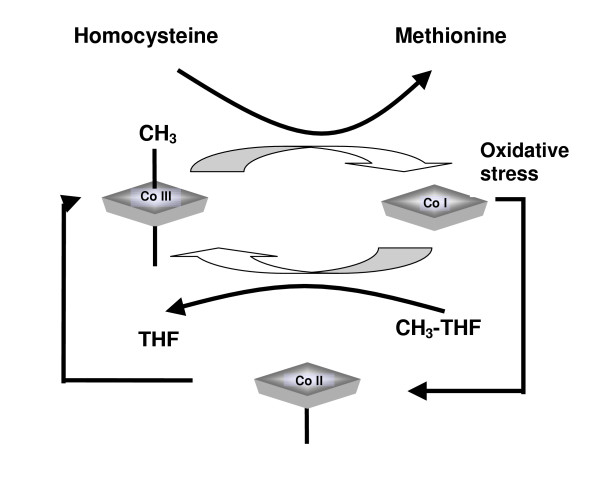
Details of the methionine synthase reaction: Homocysteine reacts with the methyl group of methionine synthase-bound methyl-B_12 _to produce methionine and an unstable intermediate form of B_12_, cob(I)alamin. Cob(I)alamin then reacts with methyl-folate (CH_3_-THF) to generate free folate (THF) and regenerate methyl-B_12_. The vitamin shuttles between methyl-B_12 _and cob(I)alamin states. Cob(I)alamin is occasionally deactivated by oxidation to cob(II)alamin. Reductive remethylation of Cob(II)alamin requires a methyl group donated by S-adenosylmethionine. Deactivation usually occurs every few hundred cycles, but AD and age-related oxidative stress might augment this process [23].

**Figure 4:**
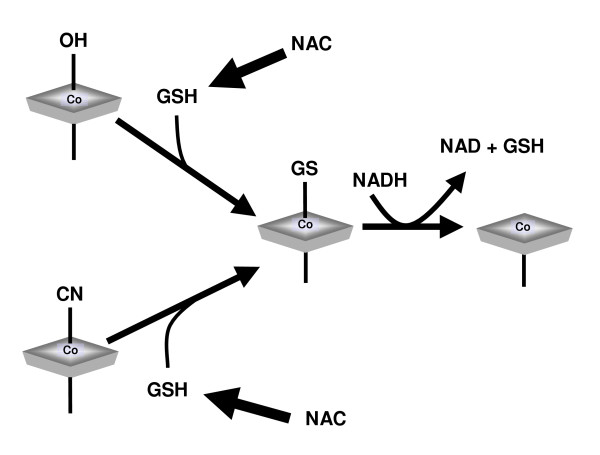
NAC and intracellular B_12 _processing: Potential role of the glutathione precursor NAC in intracellular vitamin B_12 _metabolism (GSH = reduced glutathione).

NAC might act as an antioxidant, reducing the effects of oxidative stress on the methionine synthase reaction [33] (Figure 3). Additionally, it might facilitate intracellular vitamin B_12 _processing by increasing levels of reduced glutathione (Figure 4). Reduced glutathione is required for the conversion of cyano-B_12 _and hydroxo-B_12 _to glutathionyl-B_12_, prior to reduction to the enzymatically active forms of the vitamin by cobalamin reductase [34]. NAC also increases urinary excretion of homocysteine, leading Ventura *et al *to suggest that this approach may be an important associative or alternative therapy for hyperhomocysteinaemia [35].

The radiological findings in Case 7 are notable. Elevated homocysteine levels are associated with brain atrophy and white matter lesions [6,36,37]. The annual estimate of progression of white matter lesions in cognitively intact elderly individuals is approximately 0.6 mL/year [38,39]. Though not formally quantified, the apparent halting of disease progression in Case 7 is of significance. Follow-up scans were not performed in the earlier cases.

A notable feature in all cases was the complete absence of anaemia. Indeed, despite the clear association between metabolic evidence of B_12 _and/or folate deficiency and dementia, anaemia and macrocytosis are invariably absent in these patients [40]. The dissociation between the neuropsychiatric and haematological features of these deficiencies suggests that they may not always share a common pathogenesis. Hyperhomocysteinaemia in these patients potentially arises from oxidative *depletion *of vitamin B_12 _and folate [23,41]. Such depletion is, of course, subtly different from our current concepts of classical deficiency due to malnutrition or malabsorption.

The epidemiological evidence is now of sufficient strength that elevated levels of homocysteine should be considered a *potential *risk factor for dementia in elderly patients [42]. At the very least, clinicians should determine the folate and vitamin B_12 _status of these patients, irrespective of whether or not there is a macrocytic anaemia.

Randomized controlled clinical trials are required to formally evaluate the apparent beneficial effects of this synergistic approach to cognitively impaired hyperhomocysteinaemic patients. However, these initial studies suggest that disturbances of vitamin B_12 _and folate metabolism may indeed be an important remediable factor in the aetiology of these fascinating but devastating diseases.

## Competing interests

I am a Scientific Advisor and shareholder of *COBALZ *Limited – a UK based company developing alternative oral formulations of vitamin B_12_, including formulations with NAC as an additional compound.
